# Current Status and Applications of Adaptive Laboratory Evolution in Industrial Microorganisms

**DOI:** 10.4014/jmb.2003.03072

**Published:** 2020-05-08

**Authors:** SuRin Lee, Pil Kim

**Affiliations:** Department of Biotechnology, the Catholic University of Korea, Gyeonggi 14662, Republic of Korea

**Keywords:** Industrial strain development, adaptive laboratory evolution, systems metabolic engineering

## Abstract

Adaptive laboratory evolution (ALE) is an evolutionary engineering approach in artificial conditions that improves organisms through the imitation of natural evolution. Due to the development of multi-level omics technologies in recent decades, ALE can be performed for various purposes at the laboratory level. This review delineates the basics of the experimental design of ALE based on several ALE studies of industrial microbial strains and updates current strategies combined with progressed metabolic engineering, *in silico* modeling and automation to maximize the evolution efficiency. Moreover, the review sheds light on the applicability of ALE as a strain development approach that complies with non-recombinant preferences in various food industries. Overall, recent progress in the utilization of ALE for strain development leading to successful industrialization is discussed.

## Introduction

As microorganisms are repeatedly subcultured in the laboratories, the lag phase is shortened and the growth rate is increased compared to the initial culture. This is the result of “struggling for existence”, as Darwin stated. During the process of competing in an environment with limited resource, adaptive mutations are passed down, which bring about changes in the whole population. In this way, unintended evolution occurs over a short period. Adaptive laboratory evolution (ALE) is a narrow-experimental evolution that mimics this natural phenomenon in laboratory and derives the desired phenotype. It is possible to change the environment by applying artificial selection pressure and obtain the ameliorated organism generated by the accumulation of beneficial mutations via natural selection [1, 2]. ALE was first used by the evolutionary scientist Dallinger in the seven-year high-temperature adaptation experiment [3] and has since been applied to studies on various organisms, from microalgae, mammalian cells, and viruses, to standard model organisms such as *E. coli* and yeast [4-9].

Efficient rational engineering of cellular metabolism is possible only if comprehensive knowledge of metabolic pathways is acquired; however, this aspect is elusive even in the most well-characterized model organism, *E. coli*. As coenzymes such as ATP, NADH, and NADPH are used in multiple metabolic reactions in common, complex interactions between metabolic reactions frequently impede strain improvement, resulting in different metabolic outcomes (*e.g.*, sub-optimal growth, lower product concentration) [6, 10]. Besides, practical difficulties arise when the genetic manipulation itself is complicated by polyploidy, gene essentiality, *etc*. ALE can overcome these limitations because it does not require prior knowledge of the genotype-phenotype relationship and is easy to implement practically. In particular, it is advantageous for inducing a counter-intuitive phenotype spanning numerous intracellular pathways such as diverse stress tolerances and rapid growth in specific environments. ALE has been used as a powerful complement to metabolic engineering by subsequently re-optimizing the cellular fitness of crippled recombinants [11, 12]. In addition to the strain improvement, the omics approach for the analysis of causative mutations in growth-improved strains is used for expanding intracellular regulatory networks by revealing underlying mechanisms that regulate cell metabolism [6,13-1^6^]. For example, transcriptome analysis of evolved *E. coli* with minimal genome showed that increasing the Entner–Doudoroff pathway flux, which enables efficient glucose utilization and increased intracellular reducing power, contributed to rapid growth [12]. Thus, ALE provides a straightforward reverse engineering approach that can overcome the shortcomings of existing rational metabolic engineering [2, 17].

Industrial microorganisms have long been exploited as key producers in almost every field, including food, pharmaceuticals, and other value-added chemical production. Efforts to eliminate obstacles and maximize productivity in bio-based processes are still ongoing, and ALE is one of the most effective approaches for this endeavor. In particular, microorganisms have the advantages like easy control of culture conditions, short generation time, easy manufacturing, and storage of living fossil records for each period [18]; therefore, research on microorganisms utilizing ALE has been actively conducted. Recently, a multi-disciplinary approach termed “systems metabolic engineering”, which encompasses systems biology, synthetic biology, and evolutionary engineering with the existing metabolic engineering approaches, has paved the way for the industrial use of microbial ALE.

This review presents recent ALE studies on industrial microorganisms, major considerations for efficient experimental design, and highlights the utility of various tools and strategies for strain optimization.

## Empirical Studies of ALE for Industrial Application 

The development of industrial strains aims to maximize productivity (product formation in unit time) and yield (product formation per substrate consumption) [19]. For industrial process efficiency, ALE of an industrial host can contribute to cut down the cycle time and improve product titer, strain tolerance, and substrate utilization ability. Application of ALE for strain improvement is typically categorized as 1) optimization of growth on a specific nutrient and induction of prototrophy, 2) growing under harsh conditions (non-optimal pH and temperature) or tolerating adverse environment like inhibitors generated during the process, 3) production of new substances or increment of product titer.

Empirical ALE studies aimed for improved productivity of value-added products and substrate flexibility of strains were listed to identify the preferred production strain, the evolutionary duration, and conditions for each product. These include examples of *E. coli*, *S. cerevisiae*, *C. glutamicum*, microalgae, and *Streptomyces* sp., used for increasing production of metabolites and recombinant proteins that can be used in the pharmaceutical, food, bioplastic, and biofuel industries [8,20-43]. The cases of adaptive evolution of *E. coli*, *C. glutamicum*, *S. cerevisiae*, and *G. oxidans* that enables economical bioconversion process by maximizing the utilization of relatively inexpensive substrate such as whey and biorenewable resources were also included. [9,44-51]. Some detailed examples are discussed in the following sections.

## Practical Considerations of ALE

There are several factors that the researcher should consider for successful ALE results. First, the mode of cultivation should be determined by considering the characteristics of the host strain, available man-power, and other costs incurred during the process. Batch and continuous culture are the two main methods of culturing microorganisms. As described in the review by Sandberg *et al*. [52], most of the ALE were performed using batch culture, which is simple to execute and requires no special apparatus, thus incurring less expenses. In addition, it is possible to observe a common mutation by reducing the culture volume to a small scale, such as 96 wells, or by increasing the number of replicates through automation [53]. This makes it possible to clearly identify the direction of evolution in a certain environment. However, the population density varies from flask to flask and the fluidity of the physico-chemical environment, including pH and dissolved oxygen, is difficult to keep constant due to changes in cell density that depend on the residual amount of nutrients [54]. This may lead to erroneous evolutionary pressure, which causes an undesired phenotype and makes it difficult to identify the relationship between the genotype and phenotype [55]. However, continuous culture maintains consistent environmental conditions, supplies nutrients, and manages to keep the cell density and growth rate constant [54-56]. There are also some disadvantages to this method. Since it has a long running time, it is difficult to maintain sterility and the stability of metabolic engineered strains, although mutation is helpful in terms of evolution [57]. There is also the possibility of biased evolution of mutants that have adhesive properties to prevent washing out [58]. Most of all, it is expensive to set up and operate, and it is hard to conduct in parallel. To reduce the cost and enable parallel operation, while taking advantage of continuous culture, miniaturization of the vessel can be an alternative route [59].

Second, it is essential to apply the appropriate selection pressure to screen the desired phenotype from the entire population. For the purpose of increasing the growth rate of organisms, maximizing the use of alternative carbon sources, harsh conditions (non-optimal pH and temperature), or tolerance for substances generated during production, the organisms favorable for survival in specific conditions reproduce rapidly and the desired phenotype and growth rate are coupled, facilitating screening. For example, overproducer of antimicrobial substances can be easily identified by superior cell viability in hostile environment [39, 40]. However, the phenotype is sometimes not linked to an improvement in growth rate. Overproduction of growth-irrelevant metabolite or an evolution-induced altered mutation acts as a metabolic burden thus reducing the growth rate or biomass [60]. Therefore, metabolic pathways are adjusted to couple desired phenotypes to growth for growth-based selection. This method of applying artificial evolutionary pressure by metabolic engineering was proposed with the name “metabolic engineering-guided evolution (MGE)” [61]. Considerable numbers of the studies presented in Table 1 started with a mutant rather than a wild type strain, or a genetically modified mid-point strain, to prevent efflux of resources that interfere with production or to exert evolutionary pressure. However, in the process of MGE, prior knowledge for rational design is required, making its application limited to targeting well-known materials; it also may require complicated manipulation. In addition to MGE, as an alternative to screening growth-uncoupled phenotype, the transcription factor-based or riboswitch-based biosensors has recently emerged as detectors of intracellular substances not related to growth [27, 29, 62]. In the ALE study of *C. glutamicum* engineered for the production of L-valine, which was the first to propose this method [29], the cells expressing the downstream green fluorescent protein (GFP) reporter gene depending on whether the transcription factor (Lrp) binds to the promoter were sorted by fluorescence-activated cell sorting (FACS). The group showing strong fluorescence was isolated and cultivated in an iterative manner to induce evolution. In another instance, Choi *et al*. reported that it is possible to increase the production of a recombinant protein by inducing a *parB* mutation that increases the copy number of the plasmid in the cell by sorting and passaging cells with increased GFP expression using FACS [43]. As such, the usability of biosensor-based high-throughput screening that enables straightforward and rapid screening is expected to increase in the future.

The third is the time span, that is, when to stop the ALE and obtain the final evolution product. Determining the end point is entirely up to the researcher's decision. Generally, due to the tendency for most causal mutations to fix early, beneficial mutation rates tend to decrease with longer evolutionary periods [63], although this does not mean that organisms stop adaptive evolution. A generation is generally the scale used to indicate the period of evolution, and days and transferred numbers are also displayed depending on the experimental method. Because of ambiguous criteria, it is difficult to compare experiments for evolutionary periods, even when the experiments evolve the same strain. For this reason, cumulative cell divisions (CCDs) have been proposed as a new time scale. CCD utilizes the total cumulative number of the whole population, which is substantially proportional to the mutation rate, as a denominator so that it can describe an accurate quantitative criterion for ALE’s execution time [64].

Successful ALE results depend on the heterogeneity of the population resulting from mutations [19, 64]. Although artificial mutagenesis may induce mutation of the parent strain to increase diversity, adjusting the passage size (the amount of transferred cells) that can act as a bottleneck during serial transfer can also affect diversity [52]. It is also important to keep the cell state constant for each cycle. In addition, considering that the growth rate increases while the fitness increase decreases as the culture is prolonged, it seems trivial, but critical, to optimize the passage method, such as gradually increasing the passage frequency and reducing the passage size.

## Exploiting Genetic Engineering Tools to Assist Evolution

ALE can be accompanied by genetic engineering to reconstruct metabolic pathways or to identify causality between mutations and phenotypic changes, as analyzed by WGS of evolutionary strains [61]. Classical metabolic engineering tools such as lambda red recombinase expression vector of *E. coli* and suicide vector (pK*mobsacB*) of *C. glutamicum* are widely used to introduce simple genetic changes in evolved strains [12, 65]. The advent of the CRISPR (Clustered Regularly Interspaced Palindromic Repeats)-Cas (CRISPR-associated proteins) system, recognized as a new genome editing tool, has made manipulating genes easier and more sophisticated. In addition to direct genome disruptions such as point mutations, deletions, and insertions, combinatorial methods that use improved Cas proteins (dCas9) or even other fused enzymes, such as CRISPR interference (CRISPRi) [66] and CRISPR activation (CRISPRa) [67], have been developed to allow metabolic flux control. Recently, a prime editor that fuses transcriptase was developed [68], and the scope of various recombinant productions is being expanded.

The mutation rate in ALE is accelerated by population heterogeneity [69]. Therefore, to secure genetically diverse libraries, random mutagenesis of whole cells using mutagens, such as N‐methyl-N'-nitro-N-nitrosoguanidine, ethyl methane sulfonate, can be performed using the same principle as the classical in vitro directed evolution [33, 70, 71]. However, the mutation rate increases in case of traditional in vivo mutagenesis, while the efficiency may decrease because of hitch-hiking (neutral, deleterious) and non-target mutations. These limitations can be overcome by targeted in vivo mutagenesis. Typical targeted in vivo mutagenesis includes an orthogonal replication pair (vector and DNA polymerase), ColE1/Pol [72], OrthoRep [73], and Muta T7 [74], which are composed of a phage-derived promoter, RNA polymerase, and DNA-damaging enzyme (cytidine deaminase). Because the characteristics of each method (applicable species and mutation introduction method) are different, it is important to use them properly according to the specific purpose. Furthermore, mutagenesis of the target sequence can be achieved using modified CRISPR-Cas technology such as EvolvR [75], CasPER [76], CREATE [77].

As to altering complex phenotypes, genome evolution approaches including genome shuffling [78], global transcription machinery engineering (gTME) [79], and MAGE (multiplex automated genome engineering) [80] are suitable. Sandberg *et al*. introduced several combinations of mutations in the starting strain using MAGE in the study of thermotolerant *E. coli*, enabling more efficient causative mutations and epistatic interactions [81]. Recently, novel in vivo continuous mutagenesis, genome replication engineering-assisted continuous evolution (GREACE) and feedback-regulated evolution of phenotype (FREP), which can generate mutations during evolution, have been developed. GREACE enables mutagenesis coupled with selection during evolution using DnaQ (the proofreading element of DNA polymerase) mutant library [82]. Luan *et al*., who first introduced this system, developed *n*-butanol, acetate-tolerant *E. coli* for biofuel production. Subsequently, Wang *et al*. increased the titer and yield of *E. coli* lysine by 14.8% and 9.3% using GREACE [83]. FREP is a synthetic gene circuit that detects product concentrations in vivo and regulates the mutation rate of the genome [37]. Chou *et al*., the inventors of FREP, increased tyrosine and lycopene yields in *E. coli* by approximately 5 and 3 times after 24 h, respectively.

As these approaches are capable of mutation control, they can compensate for the disadvantages of the existing random mutagenesis, such as an unstable genotype and nonhomogeneous phenotype of the final strain; thus, they could be promising strategies for industrial strain development.

## ALE for Developing Non-Recombinant Strains 

Productivity and product quality may be degraded due to environments that are not suitable for growth, such as depletion of nutrients, low oxygen, and high osmolarity, which can occur on an industrial scale [84, 85]. Therefore, as stated above, ALE is often accompanied by genetic manipulation. However, unlike the production of fine chemicals without genetic materials, genetically modified microorganisms (GMMs) are rarely commercialized in traditional food and probiotics industries [86]. Apart from the definition of food-grade genetically modified organisms (GMOs) and the enactment of related laws, the production of GMO-derived food has been a constant debate due to the combination of negative consumer perceptions and distrust of conglomerates [87]. The use of GMMs, mostly composed of lactic acid bacteria and yeast, is disinclined because of the possibility of viable GMOs in the product to colonize during the digestive period in the human body, even if they pass through the stomach [88]. Although two genetically modified *S. cerevisiae* strains have been approved, the proportion of revenue they earn in the real market is expected to be negligible, and it is expected that it will take a long time for GMMs to become universal and affect the market [89].

Therefore, fermentation strains have been ameliorated through classical methods, such as random mutagenesis, and natural conjugation and transformation [90]. These methods are not subject to the strict regulations related to GMOs, but have the disadvantage that it is difficult to express brand-new characteristics not inherent in the strain. Furthermore, in the case of brewing yeast, sexual hybridization is intricate due to the loss of the spore-forming ability and sexual reproduction caused by industrial domestication [84, 91, 92], and random mutagenesis is also limited because of the complex genome structure of the production strain. This may also lead to the loss of the desired phenotype [89]. Experimental evolution is drawing attention as an efficient and non-genetic engineering technique that can solve these problems. In adaptive evolution, strain aneuploidy and polyploidy are not a problem [93, 94]. Additionally, there is no need for the complicated work of screening strains of the desired phenotype and prior knowledge of genes involved in the attribute is not required [84]. For this reason, in the case of strains used in fermentation or probiotics, it is advantageous to improve varieties through ALE rather than genetic manipulation for commercialization.

However, there are limits to improvements without genetic modification. Several yeast studies have produced plant secondary metabolites through heterologous expression [95, 96], but relying only on evolution without external DNA sources cannot drive non-intrinsic pathways, thus limiting the development and production of new food flavors. This may lead to serious defects that do not lower unit costs. Another trepidation when using non-recombinant is the difficulty in identifying the source of the production strain. A unique strain is the valuable original technology of the company. However, the non-recombinant strain is naturally derived from the ancestral wild type, and it can be difficult to distinguish clearly because there is no characteristic factor to distinguish the strain due to the absence of an artificial marker, which might lead a conflict of interests between industries. However, despite these shortcomings, at this point, where the GMO controversy has not easily faded for decades, the improvement of production strains using experimental evolution without the influx of external DNA is anticipated to be used as a reasonable and practical strategy to ensure stable profits.

## A Genome-Scale Metabolic Model for Insight into Integrated Cellular Systems 

The optimization of cell factories is an iterative process (design-build-test-learn [DBTL] cycle) consisting of strain construction, evaluation, and data analysis to obtain the desired product [97, 98]. In the process of constructing an overproducer, the collaboration of ALE with the genome-scale metabolic model (GEM) [10], one of several mathematical models describing metabolic processes, facilitates obtaining a desirable phenotype [99]. GEM is an *in silico* model derived from genome-scale network reconstruction (GENRE) based on an organism-specific biochemical, genetic, and genomic (BiGG) knowledge base, which converts network construction into mathematical forms to computationally assess phenotypic properties [100]. Beginning with *Haemophilus influenza* [101], GEMs of various species have been constructed to date, and after repeated development, a more sophisticated level of model prediction is now possible (Table 2) [102]. These models contain information on gene products involved in metabolic reactions, as well as stoichiometric information of all metabolic reactions in cells; hence the entire network of life [10, 63]. Therefore, GEMs show the correlation between genotype and phenotype and can be used to predict physiological changes, such as cellular redox state or energy metabolism according to the theoretical maximum yield of product and genome perturbation. By using GEM, Otero *et al*. made it possible to guide the experimental direction by predicting *in silico* gene deletion in evolutionary engineering combining *S. cerevisiae* biomass and succinate production [26]. They identified the target gene to be deleted using the OptGene algorithm to couple the objective function (improved succinate production) and biomass. In order to prevent succinate consumption within the TCA cycle and serine synthesis metabolism flux from glycolysis, *sdh3, ser3, ser33* were knocked out, and the subsequent ALE finally yielded a 7.7-fold increase in succinate yield glycine prototroph. Likewise, in the study of adaptation against substrate alteration by Sandberg *et al*., it could be explained why the specialist subpopulation arises on glucose/acetate switching, based on discrepancy in reaction fluxes through the simulation of individual metabolic fluxes [103].

Furthermore, GEM and ALE can also be used together to derive the desired phenotype. In contrast with *in silico* experimental predictions, the desired outcome is often elusive to attain (*e.g.*, high-yield production) in vivo, due to the high degree of connectivity between diverse pathways within the cellular system and not considering a large number of regulatory constraints in GEM [10]. Through the ALE experiment, the latent potential of the production host can be realized, and the knowledge obtained from the results of omics data analysis can be used to refine the design again to complement the results. Ibarra *et al*. performed ALE to resolve inconsistencies between *in silico* prediction and actual experimental results of *E. coli* growth rate when glycerol was provided as the sole carbon source, enabling the yield of theoretical optimal growth results [11]. In recent years, since attempts have been made to integrate not only metabolic processes in GEM, but also information about proteins (structure, synthesis, and secretion pathways), prediction through modeling is expected to be more accurate [10, 19].

## Automation of ALE

In the process of manual cultivation for a long time, several practical problems arise. First, it is very laborious to continuously and frequently monitor cells during long-term experiments. Since most ALEs are performed for increasing growth rates, the monitoring intervals should be shorter as cells evolve to accurately measure growth rates; however, there are limitations to such manual approach. In addition, the batch culture used in many ALE studies is more strenuous and error-prone because additional manual processes, such as periodic passage, are required. These problems can be dealt with by the introduction of full or partial automation. For example, using a machine, the passage of batch culture can be performed at a frequency 3-7 times higher than the manual process, so it is not restricted by the experimenter's schedule, allowing passage at a constant growth stage and reducing fluctuation of passage size. This can prevent beneficial mutation loss [104, 105]. The problem of maintaining exponential growth and the appropriate selective pressure level can also be solved, unwanted effects caused by manual adjustment can be minimized, and genetic diversity can be secured, resulting in a shorter time to obtain an endpoint. In addition, it is possible to consolidate statistical significance by increasing the number of independent replicates beyond the capacity of a person to perform [16], readily introducing changes in the culture environment (substrate gradients, temperature, etc.) and monitoring the status in real time. Accordingly, ALE studies that automate repetitive batch cultivation processes have been continuously reported [59,81,106-108]. By measuring the optical density (OD), cell growth status can be checked and then automatically passed to the next vessel at the right time to take advantage of the aforementioned, as well as to measure the pH and dissolved oxygen in real-time to monitor the immediate response of the cells to the environment. There are also types of machinery that enhance the automatic aspect of continuous culture. An morbidostat can detect a population increase and adjust the levels of chemicals acting as the selection pressure, as feedback. A fluorostat, which combines fluorescence detection with a turbidostat, can be applied to determine gene expression levels [109-111]. Recently, eVOLVER, a framework consisting of open-source software and flexible hardware that can customize experimental conditions according to a specific purpose, was developed. It was introduced as a tool that can be reconfigured in the laboratory, and a detailed user guide to operate this platform is provided [112, 113]. High-throughput cultivation techniques can be used to derive the outcome of experimental evolution more quickly and accurately, and the acquired knowledge and outcomes can be used industrially, such as improved understanding of bioprocessing and the resulting improved strains. However, considering that most of the automated systems introduced above are laboratory scales of μL and mL, verification through scale-up is required.

## Conclusion

**Fig. 1:**
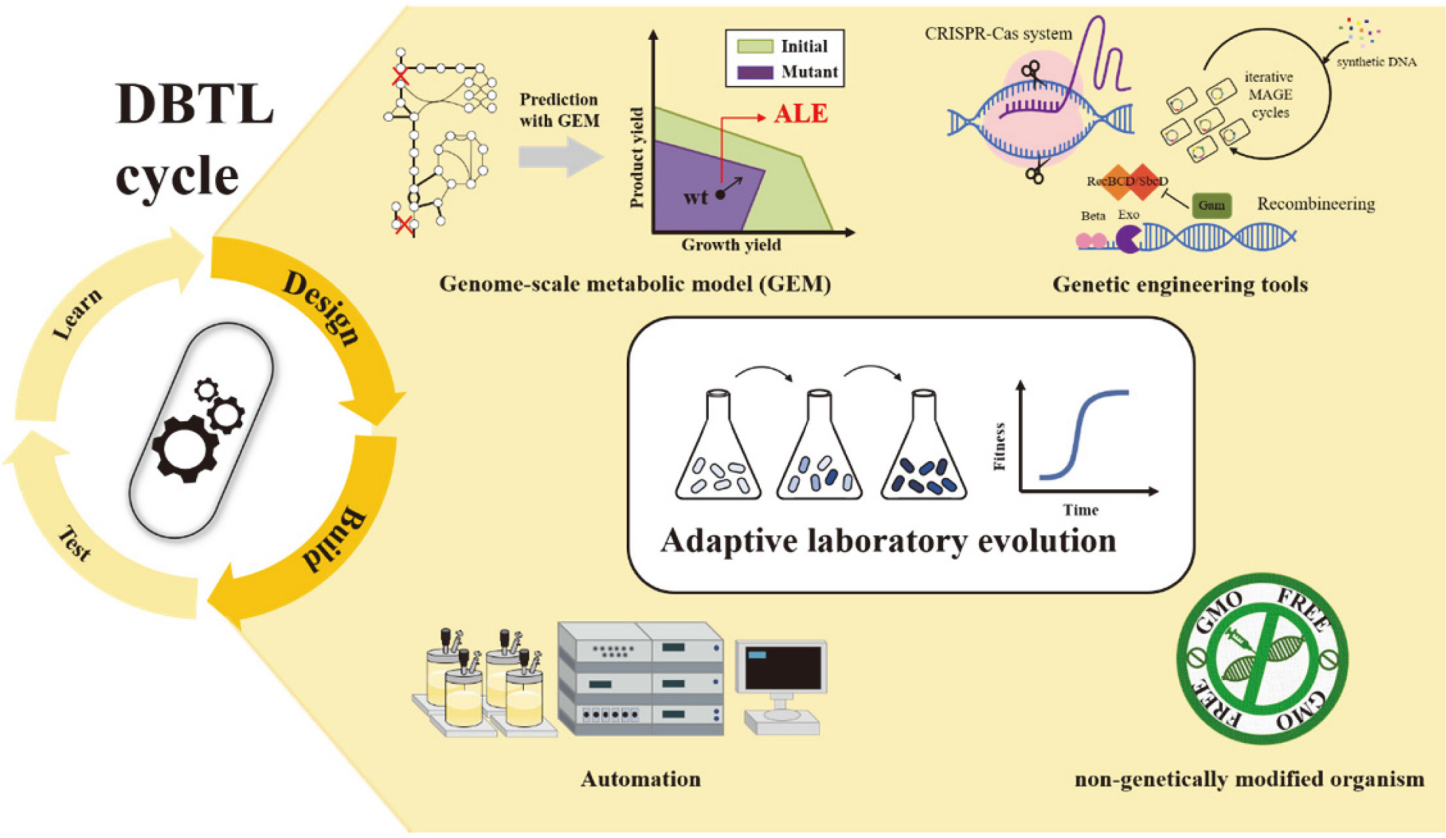
Recent progress in tools and strategies for ALE. Adaptive laboratory evolution (ALE) is one of the most preferred approaches to optimize host in the design-build-test-learn (DBTL) cycle. It elicits desired outcome efficiently along with genome-scale metabolic model, genetic engineering tools and computational technology as well as aids the amelioration of non-recombinant strain in the food industry. CRISPR-Cas system, Clustered Regularly Interspaced Palindromic Repeats- CRISPR-associated proteins system; MAGE, multiplex automated genomic engineering.

In conclusion, connecting a theoretical study directly to the success of industrialization remains a challenging task. The fragmentary knowledge of host metabolic pathways is insufficient to predict the downstream effects caused by the complex network in the cell. The scale of the culture and the industrial process at the laboratory level are different, which is also a major factor that hampers the practical use of the developed strain. Thus, it is inevitable that considerable trials and errors in the process of constructing a microbial cell factory are conducted. The point is that this series of processes should not just end in failure, but it is the interpretation of the results that should complement the underlying knowledge and reduce the development process itself, resulting in cost savings and industrial competitiveness. In this regard, ALE combined with systems biology and synthetic biology tools is a prominent strategy that can be used in the design and build steps of the Design-Build-Test-Learn (DBTL) cycle for microbial strain development (Fig. 1). The *in silico* metabolic model allows organic connectivity of the testing, building, testing and learning with a more in-depth analysis of the integrated cell system perspective using WGS and omics analysis data. In addition, automation and high-throughput screening of ALE have made more accurate and rapid development and detection possible. In this context, the multi-disciplinary toolboxes applicable for the efficiency of the development process will continue to be developed, and the base knowledge of various species should also be expanded so as not to be limited to a few platform species.

## Figures and Tables

**Table 1:** Adaptive laboratory evolutions of industrial microorganisms categorized by product and feedstock.

Product	Species	Condition	Span	Reference
Organic acid and amino acid				

Chirally pure lactates	*E. coli* SZ194, ∆*ldhA::ldhL-FRT*	Glucose minimal medium containing betaine	up to 120days	[20]
Carboxylic acid	*E. coli* MG1655, ∆*fadD*∆*poxB*∆*ackA-pta: cmR*	MOPS minimal medium with octanoic acid	714 h	[21]
D-lactic acid	*E. coli* W strain ∆*pflB*∆*pta*∆*adhE,* ∆*frdABCD*∆*aldA,* ∆*CscR*	LB or NBS medium	About 56 days	[22]
L-lactic acid	*E. coli* SZ470, ∆*adhE*∆*ldhA::ldhL*	LB-xylose medium	3 months	[23]
D-lactate	*E. coli* KO11 (ATCC 55124)	10% Glucose LB medium	About 12 days	[24]
L-lactic acid	*E. coli* W3110, ∆*focA-pflB::FRT*∆*frdBC*∆*adhE::FRT* *ackA::FRT*∆*ldhA::(ldhL-frt)*	GlcM9 medium	12 days	[8]
Succinic acid	*E. coli* K12 ∆*ptsG*∆*manX E. coli* K12 ∆*ptsI*	LB medium	48h	[25]
Succinic acid	*S. cerevisiae* CEN.PK113-5D, ∆*sdh3*∆*ser3*∆*ser33*	gradually reducing glycine	nd^*^	[26]
Muconic acid	*S. cerevisiae* BY4741, ∆*aro3*∆*aro4::PGPD-aro4_k229l_* *Ty2δ::P_GPD_-ECL_01944_opt_*∆*zwf1*[115]	Geneticin and 4FP (anti-metabolite) containing medium	1325 h	[27]
L-ornithin	*C. glutamicum* ATCC 13032, Δ*argF*Δ*proB*Δ*speE*	gradually reducing ornithine	70 days	[28]

Alcohol				

Ethanol	*S. cerevisiae* CEN.PK113-7D	Sucrose-limited chemostat	90 generations	[30]
Ethanol	*S. cerevisiae* BY4741 expressing xylose isomerase from *B. cenocepacia*	YNB medium containing xylose	40 passages	[31]
1-Butanol	*E. coli* JCL166, *ΔadhEΔldhAΔfrdBC*	Conversion from LB medium to M9 medium	nd^*^	[32]
Isobutanol	*E. coli* JCL16 NTG-created mutant	GlcM9 medium with norvaline (valine analog)	nd^*^	[33]

Lipid				

Lipid	*C. reinhardtii* cc4324 derivatives	Gradually reducing NH_4_Cl (nitrogen)	84 days	[34]
Lipid	*R. opacus* PD630	Gradually increasing phenol concentration	40 passages	[35]

Pigment				

Carotenoids	*D. salina*(UTEX LB #200)	Blue light stress	16 cycles (each cycle was conducted for 5 days)	[36]
Lycopene	*E. coli* MG1655 with plasmid (lycopene synthase gene, mutator module and sensor)	went through the FREP	432 h	[37]
β-carotene	*S. cerevisiae* GSY1136 strain [115]	Hydrogen peroxide stress	about 40 days	[38]

Antibacterial & antifungal agent				

Antibacterial compounds against MRSA	*S. clavuligerus* 27064	Co-culture with methicillin resistant *S. aureus*(MRSA) N315	About 90 days	[39]
Multiple antifungal agents	*S. variabilis* AFP2	Co-culture with *C. neoformans* 14116	30 days	[40]

ETC				

Dihydroxyacetone	*E. coli* strain C, Δ*ldhA::FRT*, Δ*adhE::FRT*, Δ*ackA::FRT*	NBS or AM1 medium with Glucose, gradually reducing NaOAc	>2,000 generations	[41]
Improved methyltransferases (Mtases)activity	*E. coli* JW3582, Δ*cysE* [117]	M9 medium supplemented with methionine and substrates of Mtases	nd^*^	[42]
Recombinant protein	*C. glutamicum* ATCC 13032	7-times sorting by measuring fluorescence	in 2 weeks	[43]

Utilization of alternative carbon source				

Lactose	*S. cerevisiae* NCYC869-A3/T1[118]	Semisynthetic lactose (SSlactose) medium,	>120 generations	[44]
Xylose	*S. cerevisiae* RWB 217 derived from CEN.PK102-3A [119]	Synthetic medium with Glucose and xylose	1,000h	[45]

Utilization of alternative carbon source				

Xylose	*S. cerevisiae* BY4741, Δgre3, URA::GPDp-xylA*3-CYC1t-TEFpXKS1-CYC1t, Leu:: GPDp-xylA*3-RPM1t-TEFp- tal1-CYC1t YDL236w:: His	Xylose medium	24 days	[46]
Xylose and mEthanol	*C. glutamicum* ATCC 13032, derivatives	CGXII medium with xylose and mEthanol	206 days	[47]
Glucose	*G. oxydans* 621H recombinant (*mgdh::Gm*)	Glucose medium	50 days	[9]
Glucose	*G. oxydans* strain evolved to use Glucose [9]	Glucose medium	25 days	[48]
Lactate or glycerol	*E. coli* MG1655	M9 minimal Medium with lactate or glycerol	>600 generations	[49, 50]
1,2-propanediol	*E. coli* MG1655, GC strain [120]	M9 minimal medium with L-1,2- propanediol	700 generations	[51]

*nd-no data

**Table 2:** The latest genome-scale metabolic models of industrial platform strains.

Organism	Main products/applications	Model	Description	Ref.
*E. coli* K-12 MG1655	Biofuel, multipurpose recombinant proteins	*i*ML1515	1515 genes, 2719 reactions, 1192 metabolites	[120]
*S. cerevisiae*	Alcoholic beverages, bakery products and bioethanol	Yeast8 ecYeast8 panYeast8 coreYeast8 proYeast8	1133 genes, 3949 reactions, 2680 metabolites	[121]
*C. glutamicum* ATCC13032	Amino acids	*i*CW773	773 genes, 1207 reactions, 950 metabolites	[122]
*B. subtilis*	Industrial enzymes and antibiotics	*i*Bsu1144	1144 genes, 1955 reactions, 1103 metabolites	[123]
*Alternaria* sp. MG1	Resveratrol	*i*YL1539	1539 genes, 2255 reactions, 2231 metabolites	[124]
*S. coelicolor*	Antibiotics and secondary metabolites	Sco-GEM EcSco-GEM	1777 genes, 2612 reactions, 2073 metabolites.	[125]
*C. vulgaris*	Lipids and pigments for biofuel and food supplements	*i*CZ946	946 genes, 2,294 reactions, and 1,770 metabolites	[126]
*L. mesenteroides* subsp. *cremoris* ATCC 19254	Used as a starter in food fermentation (dairy, meat, vegetable product)	*i*LM.c559	559 genes, 1088 reactions, 1129 metabolites	[127]
*L. reuteri* JCM 1112	Used as a starter in food fermentation and used in probiotic product, reuterin	Lreuteri_530	530 genes, 710 reactions, 658 metabolites	[128]
*N. salina*	Lipids and pigments for biofuel and food supplements	*i*NS934	934 genes, 2345 reactions, 1985 metabolites	[129]
*C. reinhardtii*	Biofuel	nd^*^	3726 reactions, 2436 metabolites	[130]

^*^nd-no data
